# A Wireless Monitoring Sub-nA Resolution Test Platform for Nanostructure Sensors

**DOI:** 10.3390/s130607827

**Published:** 2013-06-19

**Authors:** Chi Woong Jang, Young Tae Byun, Taikjin Lee, Deok Ha Woo, Seok Lee, Young Min Jhon

**Affiliations:** Sensor System Research Center, Korea Institute of Science and Technology, Seoul 136-791, Korea; E-Mails: cwjang@gmail.com (C.W.J.); byt427@kist.re.kr (Y.T.B.); taikjin@kist.re.kr (T.L.); dockha@kist.re.kr (D.H.W.); slee@kist.re.kr (S.L.)

**Keywords:** test platform, signal amplification and processing circuits, nanostructure sensors, carbon nanotube, remote monitoring

## Abstract

We have constructed a wireless monitoring test platform with a sub-nA resolution signal amplification/processing circuit (SAPC) and a wireless communication network to test the real-time remote monitoring of the signals from carbon nanotube (CNT) sensors. The operation characteristics of the CNT sensors can also be measured by the *I_SD_*-*V_SD_* curve with the SAPC. The SAPC signals are transmitted to a personal computer by Bluetooth communication and the signals from the computer are transmitted to smart phones by Wi-Fi communication, in such a way that the signals from the sensors can be remotely monitored through a web browser. Successful remote monitoring of signals from a CNT sensor was achieved with the wireless monitoring test platform for detection of 0.15% methanol vapor with 0.5 nA resolution and 7 Hz sampling rate.

## 1. Introduction

Since the phenomenon of the electrical conductance changes of individual semiconducting single-walled carbon nanotube (CNT) wire depending on gas molecules at room temperature was first reported by Dai [1] in 2000, CNT based chemical sensors for various gases [2–4], ions [5,6], and bio- materials [7–9] have been extensively studied.

Other nanostructure sensors characterized by the electrical conductance changes due to absorbance of target molecules onto nano-scale materials have high sensitivity and rapid detection speed, which can be developed by using graphenes [10,11], metal oxide, metal or polymeric nanowires [12,13], or other nanostructures. Also, the nanostructure sensors can be operated with low power and are highly integrable such that various target molecules can be detected by integrating various sensors or detection accuracy can be increased by integrating the same sensors on a single sensor-array chip.

The electrical characteristics and electrical conductance change of the sensors are measured by semiconductor parameter analyzers (SPA) or high-resolution current meters (HRCM), but due to the heaviness, large size, and high cost of these equipments, light, compact, and cheap measuring devices are highly required.

Recently, measurement circuits [14–16] or chips based on complementary metal-oxide semiconductors [17–20] for measuring electrical signals from nanostructure sensors have been developed, but these circuits or chips designed for limited electrical resistance range of specific sensors cannot be universally used for various kinds of nanostructure sensors with a wide range of resistance values from ∼kΩ to ∼100 MΩ. Before electrically connecting the sensor to the circuit or chip for measuring conductance change of the sensor, inspection of the operation conditions of the sensors by electrical current measurement with supply voltage variations are required, which cannot be achieved by these devices. Moreover, it is hard to confirm that the nanostructure sensors have solid electrical contact to the circuits or chips even if the sensors are properly operating.

In this paper, we have designed and fabricated signal amplification and processing circuits (SAPC) for universal characterizations of various kinds of nanostructure sensors having a wide range of resistance ranging from ∼kΩ to ∼100 MΩ, which are capable of determining normal operation of the nanostructure sensor and confirming electrical contact between the SAPC and the sensors by *I*-*V* measurements. Bluetooth communication is applied to the SAPC to transmit the signal change in real time from the sensors to a personal computer, and the web server on the computer transmits the signal to a smart phone through Wi-Fi communication to constitute a universal wireless monitoring test platform for various nanostructure sensors. The operation of the test platform was confirmed by using a CNT sensor to determine methanol vapor concentrations, and the results coincided well with those obtained with a commercial HRCM.

## 2. Experimental Section

### 2.1. Sample Preparation

**Figure 1.**
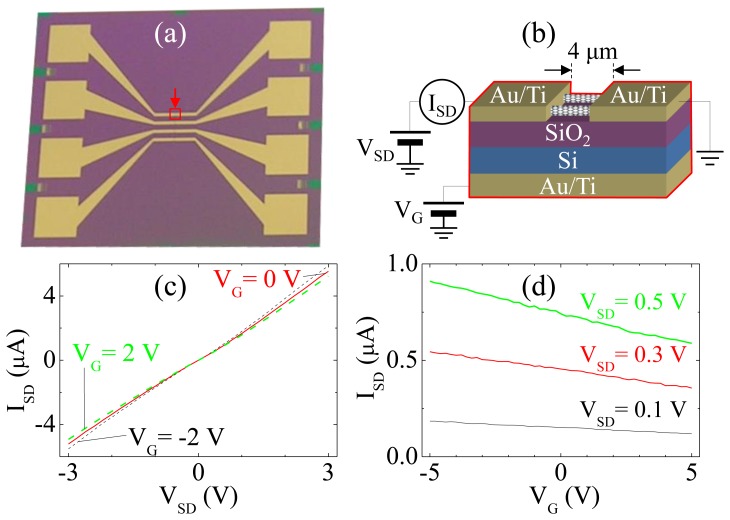
(**a**) The 4-channel CNT sensor (14 × 12 mm^2^), (**b**) schematic of electrical characteristics measurement setup (red-lined area shows the structure of the red-lined square in (a)), (**c**) *I_SD_*-*V_SD_* measurements, and (**d**) *I_SD_*-*V_G_* measurements.

Figure 1(a) shows a photograph of a 4-channel CNT sensor with four sensors integrated on a single substrate by a self-assembly monolayer method using only the photolithography process [21,22]. The 4-channel CNT sensor fabrication process begins with growing a 300-nm thick thermal oxide layer as an insulator on a boron-doped *p*-type Si wafer by a furnace (1,050 °C), after Au/Ti (10 nm/20 nm) is deposited on the back side of the substrate using electron-beam evaporation to avoid oxidation on the back side of the Si wafer after removal of the oxide layer by buffered oxide etch solution at 30 °C to form a gate electrode. Then a pre-oxygen-plasma cleaning method is applied for 40 seconds to get rid of contaminants on the insulating layer where CNTs adsorb [23], before and after photoresist (PR: AZ5214) is patterned on the insulating layer of the substrate by using only a photolithography technique to selectively adsorb the CNTs. The patterned substrate was dipped into a dispersed (30 min of sonication) CNT solution (CNT/dichlorobenzene; 0.02 mg/mL) for 3 min to adsorb the CNTs formed as a network on the exposed PR layer, and the PR pattern is removed by acetone after a rinse process (1 min) with dichlorobenzene to remove weakly adsorbed CNTs. After enhancing adhesion forces between the CNTs and the substrate by annealing on a hotplate (180 °C and 5 min) [24], both the source and the drain electrodes (Au/Ti: 100 nm/10 nm) were deposited on both ends of adsorbed CNT network by using electron-beam evaporation after another round of lithography to complete the 4-channel CNT sensors.

Figure 1(b) shows the schematic of the experimental setup, and the area in red line in Figure 1(b) is the magnification of the red area indicated by an arrow in Figure 1(a). After fabrication of the CNT sensor, the variation of electrical current between source and drain electrodes (*I_SD_*) of the CNT sensor are measured while sweeping the source-drain voltage (*V_SD_*) or the gate voltage (*V_G_*) for electrical characteristics measurements as shown in Figure 1(c,d).

### 2.2. Sub-nA Resolution Signal Amplification and Processing Circuits

**Figure 2.**
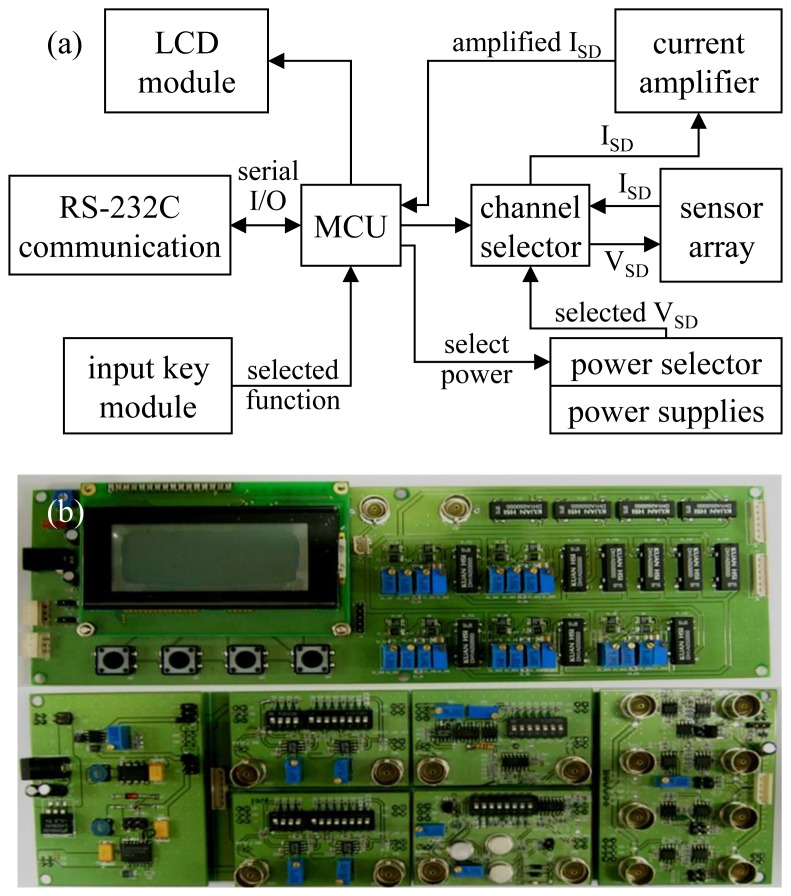
The signal amplification and processing circuits (SAPC); (**a**) block diagram and (**b**) circuit boards (250 × 80 mm^2^).

The block diagrams of the SAPC for various nanostructure sensors are shown in Figure 2(a), which consists of a liquid crystal display (LCD) module, a RS-232C module, a 4-input key module, a main control unit, five power supplies (0.1, 0.3, 0.5, 0.7, and 1 V), 13 relays (five for the power selector and eight for the channel selector for the sensor), and a current amplifier. The driving voltage of the SAPC is set to 9 V direct current to be supplied by a battery. Thus selecting the supply voltage from power supplies to the sensors at the SAPC controlled by users from user menu of the SAPC, the SAPC measures the electrical current of the 16-channel (4 × 4) sensor array while sequentially scanning the supply voltage or that of the single sensor with a single supply voltage. The current amplifier composed of two differential amplifiers can be manipulated with dip switches by users to amplify the electrical current of the sensors using 8 electrical resistors (primary amplification: 1, 10, 100, and 1,000 MΩ, secondary amplification: 0.01, 0.1, 1 and 10 MΩ) with tolerance of 1% to minimize measurement errors. The electrical signal of the nanostructure sensors is processed digitally to send the signal to the RS-232C module and the LCD module. Figure 2(b) shows the SAPC composed of two circuit boards, where the upper board of Figure 2(b) has an MCU, an LCD module, a 4-key input module, an RS-232C module, power supplies, and 13 relays and the lower circuit board has a current amplifier and a power module for driving the circuits.

### 2.3. Wireless Monitoring Test Platform

**Figure 3.**
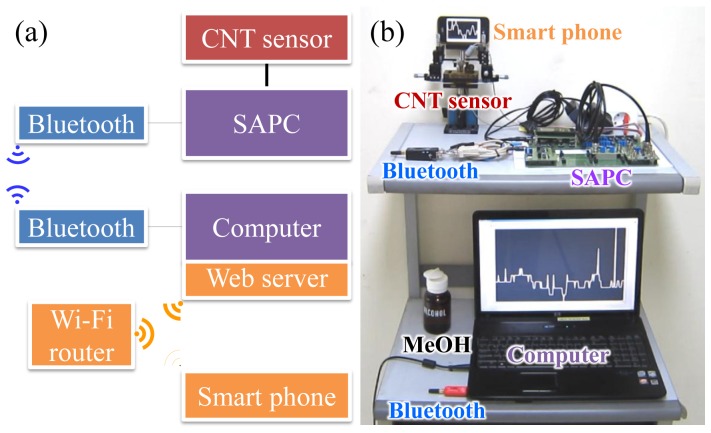
(**a**) Block diagram and (**b**) photograph of the wireless monitoring test platform.

The wireless monitoring test platform is shown in Figure 3. It consists of a sensing part, a signal processing part, a local area network part, and a wide area network part. The sensing part consists of the CNT sensors and a connector between the sensors and the SAPC. One of the signal processing parts, the SAPC, transmits the digitally processed signal from the CNT sensors to the computer via wireless local area network using Bluetooth, after amplifying and processing the electrical signal while providing supply voltage (*V_SD_*) to the CNT sensors. The computer converts the digitally processed signal of the SAPC to electrical current by LabVIEW program (National Instruments Co., Austin, TX, USA) to send it through a monitoring device. The web server is programmed with LabVIEW to monitor the electrical current of the CNT sensors by a built-in web browser of a smart phone using the wide area network for Wi-Fi communication through a Wi-Fi router.

### 2.4. Calibration Methods

**Figure 4.**
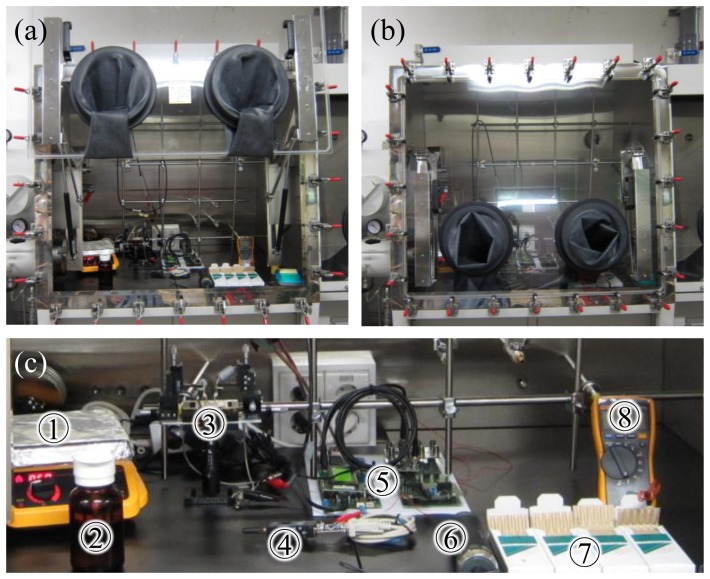
Experimental setup for detection and calibration of concentration of methanol vapor; (**a**) open glove box, (**b**) closed glove box, and (**c**) measurement devices and vapor control devices (**1**: hot plate, **2**: methanol solution, **3**: CNT sensor, **4**: Bluetooth module, **5**: SAPC, **6**: gas sampling pump, **7**: methanol detector tube, and **8**: digital thermometer).

Figure 4 shows the experimental setup for measuring various concentrations of methanol vapor using the SAPC and the CNT sensor for calibration with a commercial methanol vapor detector. All measurement devices are set in a glove box in order to maintain constant concentration of the methanol vapor. Figure 4(a,b) show the open and closed glove box. The CNT sensor, the SAPC, a thermometer, a hotplate, a methanol solution, and a commercial methanol vapor detector (gas sampling pump: GV-110S and methanol detector tube: 111, Gastec Co., Kanakawa, Japan) are emplaced inside the glove box as shown in Figure 4(c). After closing the front panel of the glove box, the methanol vapor detector in the glove box was used for calibration of the signal from the SAPC with the CNT sensor. Detection of methanol vapor was performed in a condition of atmospheric pressure with room temperature placing a bottle of the methanol solution on the hot plate (50 °C) to spread quickly and to control the methanol vapor in side of the glove box by opening and closing the lid of the bottle. Detection signal from the CNT sensor via the SAPC was sent through Bluetooth communication using the Bluetooth module (Parani-SD1000, SENA Co., Seoul, Korea) for real-time monitoring at the computer.

## 3. Results and Discussion

The *I*-*V* measurements of the CNT sensor using SPA (4155c; 10-fA resolution, Agilent, Santa Clara, CA, USA) as shown in Figure 1(c,d) prove that the CNT sensor shows the characteristics of *p*-type semiconductor such that it can be used as a chemical sensor [3].

**Figure 5.**
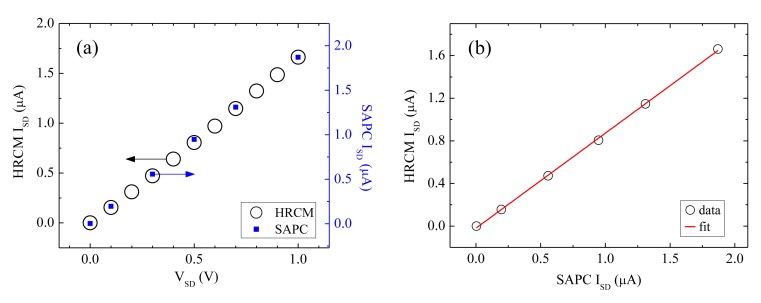
(**a**) *I_SD_*-*V_SD_* measurement of the CNT sensors by high resolution current meter (HRCM) and the SAPC and (**b**) calibration graph of *I_SD_* from the SAPC by *I_SD_* from HRCM.

The SAPC is calibrated to the HRCM (PXI-4071; 1-pA resolution, NI) by *I_SD_*-*V_SD_* measurements of the CNT sensor varying *V_SD_* from 0 to 1 V as shown in Figure 5(a). The HRCM current value against the SAPC current value obtained from Figure 5(a) fits well by using the linear least square fitting method as shown in Figure 5(b), where the fitting parameters of the slope and the intercept are 0.892 and −1.94 × 10^−8^ A, respectively and the value of the adjusted R-square is 0.999. Using this result, The SAPC current value is calibrated to the HRCM current value as shown in Figure 5(b).

**Figure 6.**
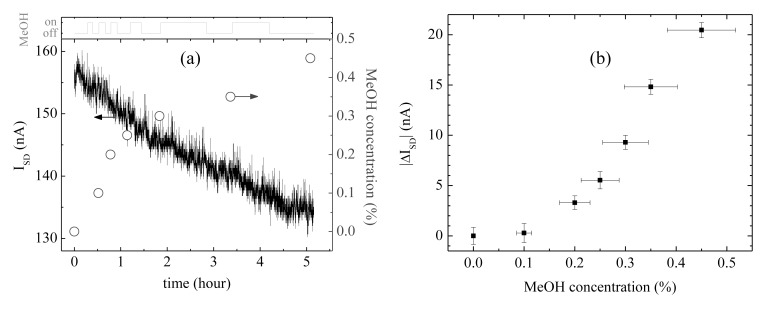
Methanol vapor detection in the glove box; (**a**) the SAPC current value of the CNT sensor and the methanol vapor concentration according to the control of the methanol vapor flow and (**b**) electrical current variation against the methanol vapor concentration.

Figure 6(a) shows the results of measuring methanol vapor concentration with the CNT sensor and with the commercial methanol vapor detector, where the methanol vapor flow was controlled by opening (‘on’ state in Figure 6(a)) or closing (‘off’ state) the lid of the bottle of methanol solution as shown in the setup of Figure 4. We waited until the electrical current value of the SAPC was stabilized before each measurement of the concentration of methanol vapor was carried out by using the commercial methanol vapor detector. The lid of the bottle of the methanol solution placed on the hotplate at 50 °C was open to diffuse methanol vapor with high velocity dispersion for a while, and was closed. By repeating the experimental methods described above, the real-time measurements of the electrical current of the SAPC and measurements of the methanol vapor concentration is shown in Figure 6(a). The temperature inside of the glove box remained almost constant around 28 °C during the entire measurement process. The conductance of the CNT sensors is known to decrease by reducing gases [25], which coincided well with our experimental results that the electrical current decreased according to the increase of the concentration of methanol vapor, which is also a reducing gas [26]. Figure 6(b) shows the concentration of the methanol vapor measured by the commercial methanol vapor detector against electrical current variation (|*ΔI*| = |*I_c_*-*I_0_*|) of the CNT sensor from Figure 6(a), where *I_c_* is the measured stabilized-electrical current value after increasing methanol vapor concentration and *I_0_* is measured stabilized-electrical current value in ambient air. The electrical current variations significantly increase with the concentration of methanol vapor.

**Figure 7.**
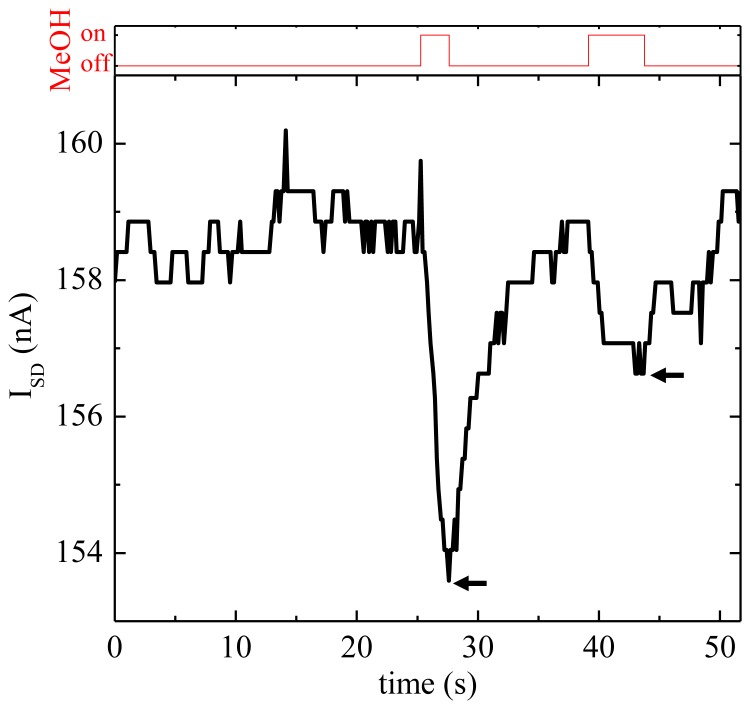
Methanol vapor detection with the SAPC and the CNT sensor in the actual field test using a cotton bud moistened with the methanol solution (the first peak for 0.25 % and the second peak for 0.15 % methanol vapor concentration indicated by the arrows).

After calibrating the electrical signal of the SAPC from the CNT sensor to the electrical current of HRCM and the methanol-vapor concentration of the commercial methanol vapor detector, the electrical current variation of the SAPC from the CNT sensor in the proximity of a cotton bud dampened with the methanol solution is shown in Figure 7, which was measured with the setup in Figure 3. Measurements were performed in a wide open space at room temperature and atmospheric pressure rather than in the glove box. Each measurement was made by bringing a cotton bud to stay for a few seconds close to the CNT sensor without contacting (‘on’ state in Figure 7) then taking it far away from the CNT sensor (‘off’state). In the same way, the experiment was repeated twice. From the experimental results of the methanol vapor detection, measurement resolution of the electrical current of the SAPC was 0.5 nA as designed. And, since the first and the second peak of methanol vapor measurement show 5.3 and 2.2 nA, the calculated concentration was 0.25 and 0.15 % from Figure 5(b).

Generally CNT sensors can measure very low concentrations (sub ppb) of gas molecules, but they have the disadvantage of having a very long recovery time to the initial conductance without high baking, UV irradiation, or vacuum pumping [3]. However, in our case of measuring high concentrations (higher than ppm), the electrical conductance of the CNT sensor was decreased by the methanol vapor and was refreshed in less than 12 seconds by the ambient air. The measured concentration of methanol vapor is less than a 40th of the flammability/explosive limits [27] of 6–36%, which shows the possibility for application of methanol monitoring and alarming (or leakage recognition) system.

## 4. Conclusions

We have constructed a wireless monitoring test platform with a sub-nA resolution SAPC and wireless network (Bluetooth and Wi-Fi communication) to test real-time remote monitoring of signals from nanostructure sensors by a smart phone. The SAPC was designed to measure wide ranges of electrical resistances from ∼kΩ to ∼100 MΩ for various kinds of nanostructure sensors, and moreover it can also determine normal operation of the sensor and confirm electrical solid contact between the sensors and SAPC by *I_SD_*-*V_SD_* measurement.

We successfully measured 0.15% methanol vapor concentration with 0.5 nA resolution and 7 Hz sampling rate in actual field test by using the wireless monitoring test platform with a 4-channel CNT sensor which we fabricated by a self-assembly monolayer method using only the photolithography process. This wireless monitoring test platform is expected to be used for various nanostructure sensors using the conductance change to detect chemicals.
